# Consistent annotation of gene expression arrays

**DOI:** 10.1186/1471-2164-11-294

**Published:** 2010-05-11

**Authors:** Benoît Ballester, Nathan Johnson, Glenn Proctor, Paul Flicek

**Affiliations:** 1 European Bioinformatics Institute (EMBL-EBI), Wellcome Trust Genome Campus, Hinxton, Cambridge, CB10 1SD, UK

## Abstract

**Background:**

Gene expression arrays are valuable and widely used tools for biomedical research. Today's commercial arrays attempt to measure the expression level of all of the genes in the genome. Effectively translating the results from the microarray into a biological interpretation requires an accurate mapping between the probesets on the array and the genes that they are targeting. Although major array manufacturers provide annotations of their gene expression arrays, the methods used by various manufacturers are different and the annotations are difficult to keep up to date in the rapidly changing world of biological sequence databases.

**Results:**

We have created a consistent microarray annotation protocol applicable to all of the major array manufacturers. We constantly keep our annotations updated with the latest Ensembl Gene predictions, and thus cross-referenced with a large number of external biomedical sequence database identifiers. We show that these annotations are accurate and address in detail reasons for the minority of probesets that cannot be annotated. Annotations are publicly accessible through the Ensembl Genome Browser and programmatically through the Ensembl Application Programming Interface. They are also seamlessly integrated into the BioMart data-mining tool and the biomaRt package of BioConductor.

**Conclusions:**

Consistent, accurate and updated gene expression array annotations remain critical for biological research. Our annotations facilitate accurate biological interpretation of gene expression profiles.

## Background

Since their introduction, microarrays have become an essential technology to measure the expression of thousands of genes in a single experiment. The widespread use of microarray technology required solutions to a number of challenges related to the analysis, storage and organisation of the data. Previous reports have detailed considerable progress in genomic scale analysis of complex gene expression data [1] the federation and warehousing of large amounts of data [2], the reproducibility of experiments across platform and laboratory [3] and the definition of standards for presenting and exchanging such data [4].

Recently, attention has been focused on improving the annotation of probes and probesets [5-7]. This has been driven by the combination of improved genome assemblies [8], more accurate genome annotations [9], new results describing the extent of transcription in the genome [10] and the recent availability of the actual DNA sequences on standard microarrays. Indeed since the time of initial probe design on most microarrays, updated genome assemblies and refined transcript structures based on data sources such as full length cDNAs, CAGE (short cap analysis of gene expression sequences) and diTags [9] have made updated probeset annotation critically important. As an example, between Ensembl release 25 http://oct2004.archive.ensembl.org and Ensembl release 51 http://nov2008.archive.ensembl.org the human genome assembly was updated twice and the number of evidence-based protein-coding transcripts identified by the Ensembl Genebuild increased by approximately 27% (from 34,111 to 46,752). This increase is especially significant considering that the number of annotated protein coding genes in Ensembl actually decreased during this same time frame (from 22,291 to 21,714). Accurate knowledge of what expression products the probes and probesets are measuring is fundamental for all downstream analysis in order to ensure accurate biological interpretation of the results.

Affymetrix GeneChips use a variable number of pairs of 25 mer oligonucleotide probes to form a probeset representing a target transcript or gene. These pairs consist of a perfect match (PM) probe identical to target transcript sequence and a mismatch (MM) probe where the 13^th ^(i.e. middle) nucleotide differs. For a given target transcript or gene, the number of PM-MM pairs varies from 11 to 20 depending on the GeneChip. Several attempts have been made to either reannotate the existing probesets or redefine probesets from the full set of probes. In both cases, these results are presented in various databases [5-7,11,12]. These previous studies are based on the same fundamental assumption that we make, namely that accurate genome annotation leads to better interpretation of gene expression data, and focus on the use of various mapping strategies to investigate ways of accurately matching probesets to the latest genomic knowledge. For example Gautier et al have reannotated the Affymetrix HG-U133A probesets by mapping the probes against Human Refseq mRNA using the BioConductor package *matchprobes *[5,13]. Most reannotation efforts addressing GeneChip microarrays have remapped probes, or target sequences to external public databases of expressed sequence. One exception to this approach is Dai et al who aligned probes to the genome of the corresponding species as well as to external references for their updated probesets definitions [7].

In this paper, we present and assess our method to map and annotate gene expression arrays, using the Affymetrix GeneChip arrays as an example. In contrast to other methods, we employ direct mappings to both the reference genome and the Ensembl gene sets as the basis for the probeset annotation. This feature makes our annotations easily accessible for use with expression of protein coding genes and also with the more complex expression patterns such as antisense or pervasive transcription that have been observed in other studies [14-16]. Moreover, the Ensembl annotations are conducted in a consistent way across all supported species, and thus do not rely on the completeness of external data resources which are necessarily less complete for some species. Ensembl is updated approximately five times each year assuring that probeset annotations incorporate the most recently released datasets.

In the following sections we (i) describe exactly how probes are mapped and assigned to transcripts, (ii) demonstrate the quality of these annotations and (iii) present the bioinformatics resources to publicly access this information.

## Results

### The mapping pipeline

**Figure 1:**
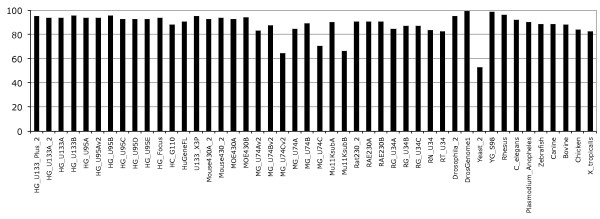
**Probes mapping to genome sequences**. The percentage corresponds to probes from the given array mapped at least once to the corresponding genome. On average about 94% of probes map the human genome, ~84% the mouse genome, ~87% the rat genome, and ~88% other genomes.

Annotation of gene expression arrays and associated probesets with an Ensembl transcript is a two-step procedure. In the first step individual probe sequences are aligned to the corresponding genome sequence and in the second step probesets having at least half of their probes matching a transcript are annotated with the associated transcript. The Ensembl analysis and annotation pipeline uses the exonerate alignment tool [17] and tolerates only 1 base pair (bp) mismatch between the probe and the genome sequence assembly. This is justified by previous work which has shown that the perfect match-mismatch (PM-MM) model is a poor model for estimation of non-specific hybridisation and PM-MM intensities should be considered and treated equally [18-20]. Descriptions of the probe to genome alignments are stored in the database as either perfect 'Full match' alignments or 'Mismatch' alignments for those probes that align with only a single base pair difference between the probe and the genome sequence assembly. Probes with more than 1 bp mismatch are not stored and not used for the second step of the pipeline. Finally, probes that align to more than 100 locations in the genome, e.g. Alu repeats, are discarded even if these are full match alignments. With these simple mapping rules most of the probes are mapped to the genome; on average 94% of probes across all human Affymetrix GeneChips are aligned to the genome with a maximum of one mismatch, the rest of the probes being excluded by one of the above mapping filters (Figure 1). Some of the Affymetrix GeneChips show a lower number of probes mapped, although these arrays are generally the "B" chips that contain probesets designed to target EST (Expressed Sequence Tag) clusters or other genes with less extensive experimental support at the time of the microarray design [21]. Indeed, using EST clusters as targets could lead to aberrant probesets, for instance ESTs from distinct genes could be falsely clustered together leading to unusual genomic mapping [22]. The alignment results give a first glimpse on the quality of the probes and provide an upper limit on the extent to which probesets can be annotated. The Additional file 1, Figure S1 describes the distribution of the number of probes per number of mappings to the genome. This distribution shows that a majority of probes have few alignments on the genome, generally one or two. Probes that map more than twice, but less than 100 times (and so pass the filter above) are more likely to be in repetitive regions (Figure S1).

It is important to note that this first step in the Ensembl probeset mapping pipeline is independent of the completeness or quality of the genome annotation, but dependent on the quality of the genome assembly. For example, Ensembl release 47 http://oct2007.archive.ensembl.org contained both an updated build of the finished *Mus musculus *assembly (NCBIm37) [23] and a combined Ensembl-Havana genebuild compared to Ensembl release 46. The new assembly alone results in some noticeable differences in the probe mapping as the number of probes mapped to the new assemblies tend to decrease, for example for the Mouse430A_2 array the number of probes mapped decreased by 8.4% (from 387341 to 354982) between the NCBIm36 and NCBIm37 assemblies. In general, for genomes with multiple assembly updates, such as human and mouse, we observe fewer probes mapping to improved assemblies, which may indicate that correcting assembly errors enhances probe mapping by removing probes designed against lower quality regions of the assembly.

### The annotation pipeline

In the second step, we associate probes and probesets from commercial arrays with Ensembl transcripts. This step uses the mapping of the individual probes by associating those probes that are part of a defined probeset to a specific transcript. For successful transcript annotation, we require that at least half of the probes of a probeset match the underlying transcript sequence and 3' untranslated regions (3' UTRs) if available. The probeset sizes are calculated dynamically using the data available from the array design as for a given array the number of probes for a distinct probeset can vary. In cases where a transcript has a 3' UTR predicted based on experimental evidence we use the transcript and we double the length of the 3' UTR sequence for the probeset annotation. If a transcript has no 3' UTR annotated we extend the 3' most exon. The lengths of the extensions are computed as the greater of either the mean or median of all 3' UTRs in the given species. In future Ensembl releases, the extension will also be limited to avoid probesets being annotated to genes in close proximity unless there is independent evidence that the UTRs overlap. These extensions of transcript sequences are used to compensate for the absence of UTRs in some of the Ensembl transcript predictions. These occur because each Ensembl transcript UTR is based on experimental evidence from cDNAs, which in some cases can be incomplete leading to shorter or missing UTRs. Affymetrix probes and microarray probes in general tend to be designed against 3' ends of transcripts [24], mostly on the basis of EST evidence from NCBI's UniGene. Since for most species in Ensembl full length cDNA sequences are limited, extending the transcript length using a species-specific UTR size is a consistent means to successfully map probe sets to Ensembl transcripts.

### Mapping probes to the Transcript set - Effects on the probeset annotation

In addition to mapping the probes to the genome, we investigated the effect of mapping the probes directly to the set of Ensembl transcripts represented as cDNAs. Mapping probes this way allowed us to assess the extent to which the probes are mapped across exon boundaries. The mapping of probes to cDNAs defined by Ensembl transcripts adds ~79,000 new alignments which corresponds to a ~2.2% increase, representing ~59,700 distinct probes (Ensembl release 51). Indeed some probes map at multiple locations on different genes, such as the probe 39540_at:245:7 from the HG_U95Av2 chip which maps to the Ensembl transcript ENST00000322357 on 19:3999238:4004988 (chromosome:start:end) as well as ENST00000332053 on 18:43810276:43820288. On the other hand some probes map to multiple transcripts of the same gene across different exon junctions, for instance the probe 61492_at:216:201 on the array HG_U95C maps to two different transcripts ENST00000366534 and ENST00000366533 from the same gene ENSG00000179397 but in slightly different locations. For the transcript ENST00000366534 the probe maps to the boundaries of exons 20 and 21 (ENSE00001441952 and ENSE00001441951) at the location of 1:242847609:242865435 and for ENST00000366533 maps to the boundaries of exons 19 and 20 (ENSE00001296059 and ENSE00001330630) at the location of 1:242840250:242865435. Other rare examples are probes mapping across three exons where the central exon is generally a short exon, for instance the probe 207136_at:1089:1005 from the array HG_U133_Plus_2 maps to the transcript ENST00000307959 on three exons, 7 bp on ENSE00001275564, 10 bp on ENSE00001425447, and 8 bp on ENSE00001275540. This 10 bp exon has strong supporting evidence from external protein identifiers P36575, NM_004312 and P36575-2 (Additional file 2, Figure S2).

**Figure 2:**
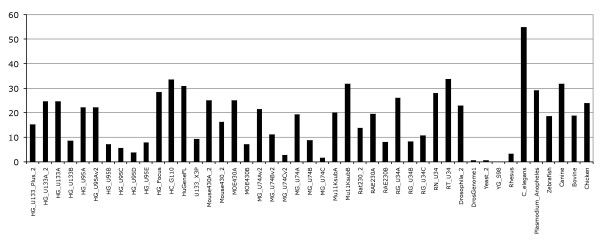
**Probes mapping to cDNA sequences**. The percentage corresponds to the number of probesets having their annotation consolidated with additional probes mapped across exon-exon boundaries in cDNA sequences.

Mapping probes to transcripts consolidates the probeset annotation description by 18% on average (Figure 2). In other words, for a given probeset more probes are mapped to the genome and used in the annotation. Typically, one or two probes map to the boundaries of exons and are used to strengthen the probeset annotation on the genome. However, the probes mapping to transcripts do not generally lead to new probesets being annotated. No additional probesets would pass required 50% transcript-level annotation rule. These results indicate that probes mapping across exon boundaries do not add new probeset annotation to transcripts but consolidate the existing annotations.

### Effect of updated array designs on probe annotation

**Figure 3:**
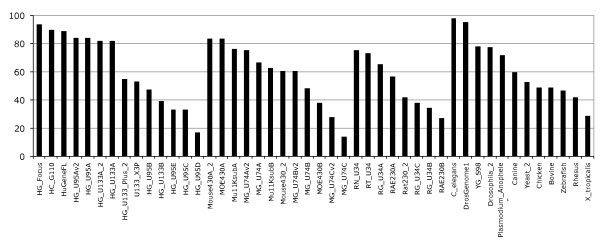
**Fraction of annotated probesets**. Percentage of annotated probesets across Affymetrix chips ranked by value and by species (Human, Mouse, Rat, Others). Note the lower percentage of annotated probesets with the "B" or "v2"chips.

The Ensembl probeset annotation pipeline is optimised to create a comprehensive gene expression array annotation based on the most current genome sequence and species-specific supporting data. To determine the quality and consistency of our annotation methods we have investigated the reasons why some probesets were not annotated by our pipeline. Using our annotation methods and the latest Ensembl genebuild, the transcript-level probeset annotation produces diverse results depending on the combination of genome sequence and GeneChip used. As seen in figure 3, the percentage of annotated probesets seems to be dependant on the probeset design quality. Indeed in the latest generation of Human expression arrays we annotate about 90% of the probesets, whereas for the "B" chips the percentage drops to about 40%. The design of the Human Genome U95 arrays were represented in 5 chips (A to E) with the A chip containing probesets designed against full-length transcripts, and the others probesets designed against ESTs from UniGene 95. Due to the evolving nature of the biological databases our results indicate that the old generation GeneChips or "B" chips have a lower annotation quality. For example, the HG_U95B/C/D/E chips have between 30% to 60% of their probesets annotated (Figure 3). Interestingly the latest Human Affymetrix chips HG_U133_plus_2 (or others v2, _2 chips) do not necessarily have the highest fraction of mapped probesets, as probesets from previous chips have been incorporated in these new arrays.

### Effect of new assemblies and genebuilds

**Figure 4:**
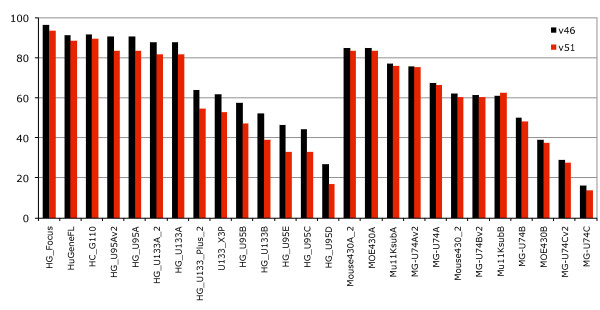
**Effect of new genebuilds and genome assembly**. Fraction of annotated probesets across Affymetrix chips for Ensembl release v46 (grey) and Ensembl v51 (red). For the Ensembl release v51 the Human genome had some genes removed due to wrong predictions, and a new assembly of the Mouse genome (NCBI m37, April 2007 strain C57BL/6J) resulted in a new genebuild.

We examined the effects a new genebuild and genome assembly could have on the probeset annotation (Figure 4) by comparing two gene sets based on the same human assembly as well as gene sets built on two versions of the mouse assembly. Both gene sets were released at the same time for Ensembl release 47 and slightly corrected for Ensembl release 51, which allowed us to attempt to separate the difference between updating a gene set on an improved genome assembly and simply updating the gene set without any underlying improvements to the genome assembly. The comparison is especially valid for human and mouse since both assemblies are now of "finished" quality and the amount of supporting information for the genebuilds such as species specific expressed sequences is similar.

The improved human genebuild focused on accurate transcript placement and UTR extensions, using both new methods and new data such as diTag information. For mouse, the new genebuild was created on the updated NCBI m37 mouse assembly. Somewhat unexpectedly our results indicate that increasing the accuracy of the transcript placement in human without a new assembly does remove some probeset annotations as some genes have had their structures improved. Between Ensembl release 46 and Ensembl release 51 the number of protein coding transcripts in human increased by about 4.8% (from 44340 to 46470), while the number of protein-coding genes decreased (from 22680 to 21714). As Dai et al described, at the time of the HG-U133 design, UniGene contained 2.8 million cDNA/ESTs but now contains 6.9 million sequences [7]. Our results show that improved assemblies have a less striking impact on the annotation than do updated gene sets as the new mouse assembly had an average 1.2% decrease in coverage across microarrays. However, this is potentially modulated by the addition for the first time in the Ensembl mouse genebuild (Ensembl release 47) of a combined gene set featuring both the Ensembl automatic gene predictions and the Havana manual annotations [25]. These manual annotations include more than 15,000 full-length protein-coding transcripts for the mouse annotated by the Havana team in addition to the Ensembl automatic genebuild. The new NCBI m37 mouse assembly is the first update of the finished mouse assembly, and has been a focus of annotation for the Consensus Coding Sequence (CCDS) project, a collaborative effort to produce a common set of CDS annotations of transcripts [26].

**Figure 5:**
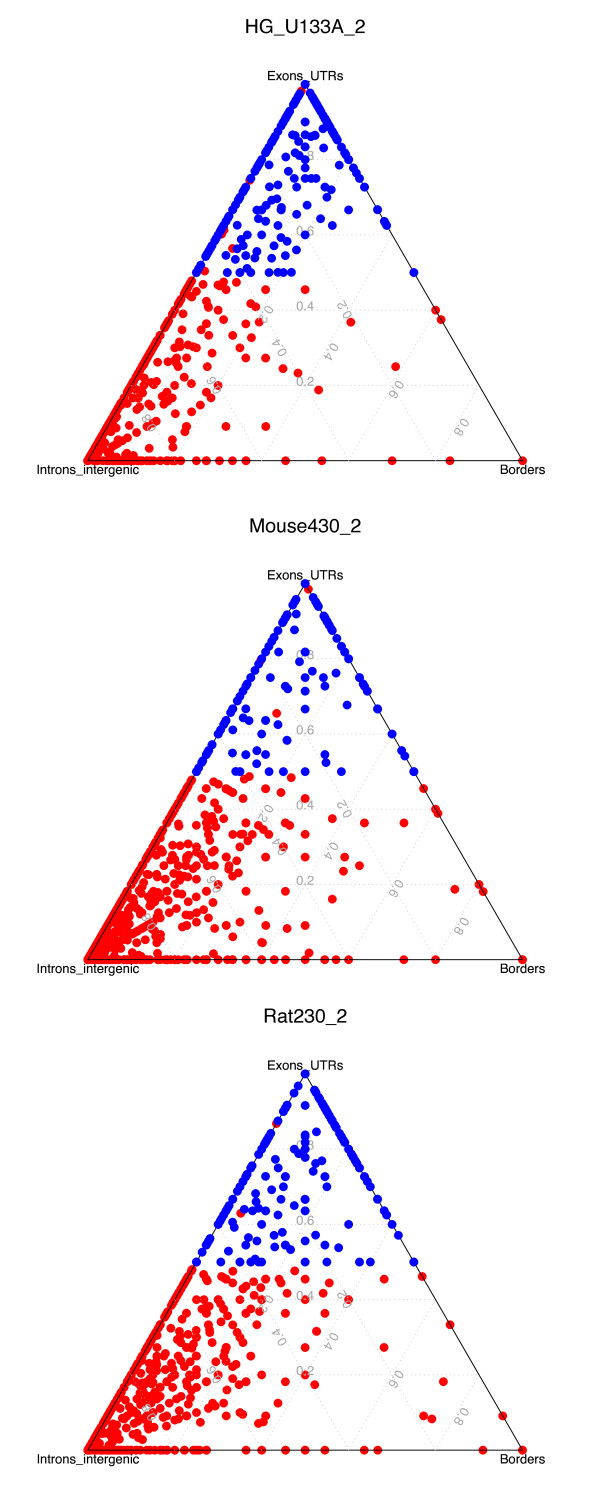
**Genomic distribution of unannotated probesets**. Ternary diagram representing the mapping of unannotated probesets for the Affymetrix gene chip HG_U133_A_2, Mouse430_2 and Rat230_2. Each corner of a plot represent a different group, the *Exons-UTRs *group with probes mapping within exons or UTRs, the *Borders *group with probes mapping to boundaries of exons/introns/UTRs/intergenic regions, and the *Introns-intergenic *group with probes mapping to introns or intergenic regions. Probesets having less than 50% of probes mapped and with 50% of those targeting exons are coloured in blue. These blue points correspond to probesets that could putatively be "annotated" if more probes where mapped to the genome. The red dots in the middle of the blue cluster correspond to probesets mapping to non protein-coding genes (rRNA, V_segment, pseudogene).

Figure 3 shows that only a fraction of probesets are not being annotated at all by our method. We investigated the mapping characteristics of the fraction of probesets that could not be annotated. Results for three representative arrays are shown in figure 5 where each dot corresponds to an unannotated probeset and the three coordinates represent the proportion of probes located in non-coding regions (intergenic, introns), coding regions (exon, UTRs), and region borders (exon junctions, intron junctions, utr junctions, and intergenic junctions) respectively. Probesets marked in blue do not have enough supporting evidence to target a transcript (50% rule) but do have at least 50% of the remaining probes mapping within coding regions. In other words these blue dot probesets do target coding regions but with too few probes to fulfil our criteria to be annotated with confidence to a transcript. These results demonstrate that the reasons for non-annotation of these probesets are consistent with the current gene annotation. Interestingly the mapping on exon-intron boundaries seems to play little role in the non annotation of probesets. Considering the Ensembl EST genes [27] did not significantly change the number of unannotated probesets (Additional file 3, Figure S3). These results demonstrate reasons why our pipeline excludes certain probesets and suggests that our methods provide comprehensive results.

### Comparing Ensembl annotation with Affymetrix annotation

Since the Affymetrix annotation is the most widely used probeset definition in numerous gene expression analysis packages including BioConductor, we compared our probesets annotation with the latest Affymetrix GeneChip annotations. The Affymetrix annotation includes information from a number of sources depending on the array including sequence information collected at the time of array design, information updated from public databases such as UniGene and protein matches from public and Affymetrix-created sources [28].

Additional file 4, Table S1 presents how Ensembl and Affymetrix compare using external accession identifiers common between these two annotations. When using the EntrezGene identifiers, the Human, Mouse and Rat chips have between 65% and 95% of EntrezGene IDs in common between Ensembl and Affymetrix annotations. These differences could be explained by the specificity of our rigorous external identifier reference mapping procedure that assigns Ensembl genes to external references. Given that ESTs from distinct genes could be falsely clustered together, some probesets could target genes with few or non-consistent external experimental references leading to separate annotations between Ensembl and Affymetrix. However comparing probeset annotation using external identifiers may not be the best approach to assess the performance of different reannotation methods when procedures for mapping external identifiers from public databases to probesets or genes are not common between Affymetrix and Ensembl. For example Affymetrix explains that UniGene identifiers are determined using the probeset's representative sequence [28]. In the case that the sequence is not found in the current UniGene database the most common UniGene identifier of the sub-cluster sequences is used. Therefore, the UniGene identifier detected using such procedure may not be what Ensembl uses in the external reference mapping procedure.

### Probesets annotated to multiple genes

Probeset annotation is complicated especially when a probeset may intentionally or unintentionally target multiple genes or transcripts. This multiple gene targeting may be the result of probesets designed against erroneous cDNA sequence or chimeric EST clusters containing ESTs from closely related genes or overlapping genes. These probesets may be more likely to exhibit cross-hybridization from splice variants or closely related genes (i.e. paralogues).

**Figure 6:**
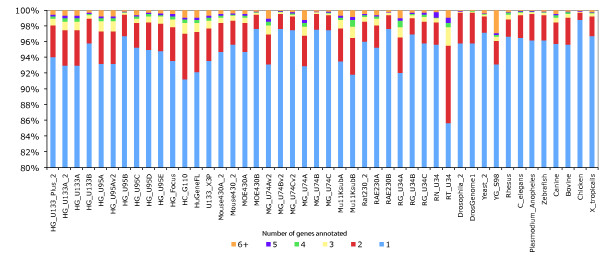
**Annotation of probesets to multiple genes**. Fraction of probesets on Affymetrix arrays that are annotated to multiple genes with each colour representing the probesets annotated to one or more genes. On average about 6.1% of probesets only target multiples genes in human, 4.6% in mouse and 5.6% in rat.

**Figure 7:**
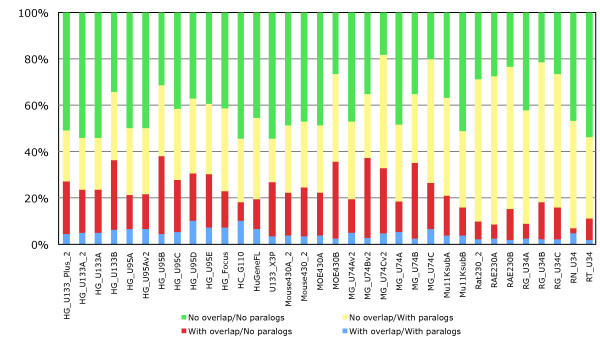
**Characteristics of probesets linked to multiple genes**. Fraction of overlapping and paralogous genes associated with probesets linked to more than one gene for the Human, Mouse and Rat GeneChips.

To establish the extent to which probesets target multiple genes, we investigated this multiple annotation problem with our pipeline. The results (Figure 6) show that on average about 6.1% of probesets target multiples genes in human, 4.6% and 5.6% in mouse and rat. Of the probesets annotated to multiple genes, the vast majority are annotated to only two genes (red bar). To investigate this we looked to see if these genes were paralogues or in close proximity to each other, or both. Our results in show that for about 60% of cases we can explain why these probesets end up annotated to multiple genes, by targeting paralogous genes or close tandem gene clusters (figure 7: average of red, yellow and blue bars). However for the remaining 40% there is no obvious biological explanation why these probesets are annotated to multiple genes, as those genes are not located in close proximity and are not paralogous (green bar). These results demonstrate that probesets targeting multiple genes can be separated in two groups, a major group targeting genes with strong sequences similarities or close proximity, and a group of probesets targeting genes without clear biological evidence.

### Allele-specific probes and mismatches

**Figure 8:**
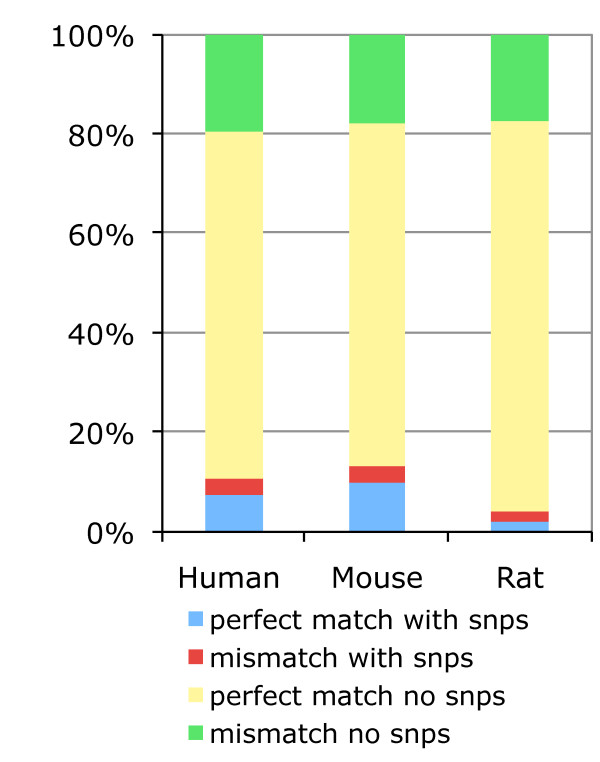
**Prevalence of allele-specific probes**. Distribution of allele-specific probes among all mapped probes for all species-specific GeneChips. In this figure the terms mismatch and perfect match correspond to the results of exonerate alignments of the probes to the genome, which have either one or no mismatchs, and not to the designations provided by the manufacturer.

Previous reports have suggested that the response of 30% to 40% of probesets on human GeneChips could be affected by single nucleotide polymorphisms (SNPs) creating allele specific probes [7]. We investigated the proportion of allele specific probes in human, mouse and rat GeneChips using the Ensembl Variation databases [29] (Figure 8). For all the probes mapped by our pipeline, and across all human, mouse and rat Affymetrix microarrays, our analysis indicates that about 11.5%, 14.3% and 4.4% respectively map at a genomic location where one or more SNPs are located (blue value). This corresponds to approximately 30.9%, 39.9% and 9.9% probesets being affected by one or more SNPs for the three species, which confirms Dai et al findings. This fraction is certain to be a lower bound on the number of probesets with potential allele specific responses as ultra high throughput sequencing technologies enable rapid genome resequencing and variation discovery [30]. Although our mapping pipeline aligns Affymetrix PM probes to the genome allowing one mismatch, all PM probes should theoretically align to the genome without a mismatch. Interestingly our results suggest that 20% of the Affymetrix PM probes hit the genome with a mismatch (figure 8: green + red values). These PM probes have the potential to represent a MM probe if the mismatch is on the 13^th ^base pair of the probe. As our pipeline allows a mismatch in the entire length of the PM probe, it may be possible that corresponding MM probes could have two mismatches. With these probes it became difficult to differentiate functional differences between MM and PM probes. For example, Harbig J et al [31] have found the presence of 206 MM probes in the HG_U133_plus_2 array that are a perfect match for some human transcript. Further work has analysed differences in PM and MM probe intensities, and some background adjustment algorithms such as RMA or GCRMA have been developed for using PM intensities only, hence omitting problems associated with MM probes [19]. Here our results provide further evidence that some PM probes include one mismatch base pair, that the corresponding MM probes could contain more than a mismatch and thus MM intensities should be carefully considered in the array background correction.

### Data access

Updated array annotations are provided as necessary with each Ensembl release [9]. Whenever a new assembly becomes available or a new gene set is produced, the probeset mapping and annotation is recomputed to present the most up-to-date and consistent probeset annotations. All gene expression array probes and probeset annotations are publicly accessible through a range of tools described below.

### Web display

**Figure 9:**
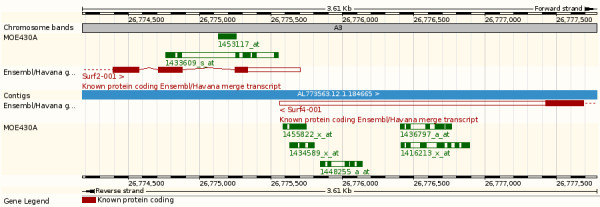
**Ensembl web display**. A view from the Ensembl genome browser showing the Mouse *Surf2*, *Surf4 *genes, and the MOE430A probesets on the chromosome 2 of the mouse NCBI m37 assembly (Ensembl v51, http://nov2008.archive.ensembl.org/)

The individual probes that are mapped to the current assembly for a given species are displayed along the genome assembly (Figure 9). If not displayed by default, microarray probeset tracks can be switched on in the "Detailed panel" tab from the configuration popup menu available from "Configure this page" link on the left panel. Clicking on a particular probe in a probe set track displays a pop-up window, which reports the 'Probe length', the 'Match length', as well as the 'Match status'. The 'FeatureView' page displays in detail the probe mapping and probeset annotation data using three panels, a karyotype panel showing the genomic locations of individual probes, an information panel with probe mapping details including genomic coordinates, mismatch status, array name and finally the probeset annotation panel with the Ensembl gene(s) and transcript(s) associated with this probeset. Finally for or more detailed information, the Gene and Transcript pages display a comprehensive list of probesets and arrays associated with the given gene/transcript.

### Data mining: Ensembl BioMart, Biomart.org and biomaRt

BioMart [32] is a data-mining tool to efficiently extract information from structured databases. Ensembl's BioMart includes the probeset annotations and is updated along with each Ensembl release. This can be used to access Ensembl genes and associated probeset annotations according to the user's specifications without any programming knowledge [33]. An example BioMart query to fetch Affymetrix GeneChip HG_U133A_2 probesets annotation is as follows:

(i) *Start*: Select the Ensembl Gene database then the species of interest, for instance Homo sapiens. (ii) *Filter*: Apply the filter "with affy hg u133a 2 ID(s): Only" in the ID LIST LIMIT panel. *(iii) Attribute*: Select the output needed, for example Ensembl Gene ID, Ensembl Transcript ID, and the name of the microarray of interest AFFY HG U133A 2. (iv) Results: Hit the button "Results" to overview of the output before final export. Not using the ID LIST LIMIT filter will output genes linked to probesets and also genes without probesets annotation.

For R/BioConductor users, the package biomaRt [34] connects to the BioMart databases to fetch the latest Ensembl annotation directly into the R console. An example of a biomaRt query to fetch Affymetrix GeneChip HG_U133A annotation would be:

>library(biomaRt)

>human_mart <- useMart("ensembl", "hsapiens_gene_ensembl")

> affy_hg_u133a_ensembl_annotation = getBM(attributes = c("affy_hg_u133a", "hgnc_symbol", "ensembl_transcript_id"), filters = "with_affy_hg_u133a", values = c("1"), mart = human_mart)

> affy_hg_u133a_ensembl_annotation [350:355,]

   affy_hg_u133a hgnc_symbol ensembl_transcript_id

350   205486_at   TESK2   ENST00000372084

351   215662_at   MMACHC   ENST00000401061

352   211774_s_at   MMACHC   ENST00000401060

353   211774_s_at   MMACHC ENST00000372082

354   219843_at   ENST00000401059

355   215903_s_at   MAST2   ENST00000372009

### The Ensembl Perl API

Ensembl maintains a comprehensive set of Perl APIs, which permit the programmatic retrieval of data from the databases. Using the API, there are different routes to access our probe mapping and probeset annotations. One is to retrieve genomic mappings using the ProbeFeatureAdaptor which will fetch all the probes aligned to a given region (slice). An Ensembl Perl API example script is available as an additional file (Additional file 5) where other methods are described. Also we store in the database the reasons why a particular probe was not considered for a transcript annotation (*e.g*. intronic, anti-sense, insufficient hits).

## Discussion

Recent studies have shown the need to regularly and automatically re-annotate gene expression probes and probesets [7,31,35]. We show that aligning probe sequences to reference genome assemblies followed by association of mapped probes to the latest Ensembl genebuild leads to precise and consistent microarray annotation across species, with differences largely due to the quality of the underlying genome sequence assembly. The strength of the Ensembl probeset annotation pipeline lies in its simple genomic mapping rule rather than mapping consensus sequences to external databases (such as RefSeq, etc.) or relating probe identifiers to public database identifiers. With our non-restrictive probe alignment procedure, we keep and show probes located in non-coding regions, but also probes and probesets mapping to multiple locations. This is done to illustrate the potentially complex nature of annotating or designing a probeset. We believe researchers should be able to access the most comprehensive set of gene expression array annotation in additional to information suggesting a probe or probeset incorrectly targets a region or another gene.

In most of the published studies, probes or reference sequences were mapped against external databases or transcripts using strict alignment rules [5,7,36]. For example, to generate reorganized probesets based on UniGene, Dai et al applied a list of seven mapping steps, including steps for removing probes mapping to multiple cDNA/EST sequences, probes mapping to more than one location on the genome, and probes mapping in non-coding regions [7]. In order to understand the problems encountered for some probesets, researchers should be able to access the quality of probe mappings for their downstream analysis.

While a majority of probesets are perfectly mapped to the genome and annotated to one gene and a few transcripts (Figure 6), a substantial number of probes and probesets present some difficulties in the mapping and annotation to transcript. In their studies Dai et al described in detail numerous problems confronted in the GeneChip probeset annotation; probeset redundancy, non-specificity of probes, genomic location issues and unreliable consensus sequences. A potential cause of these problems could be erroneous UniGene clusters used in the probeset design, for example old merged UniGene clusters. Our mapping pipeline retains genomic mappings with one mismatch regardless of the location on the genome, or multiple mappings (threshold of 100 locations). In spite of our fairly non-restrictive mapping procedure, our results reveal that not all probes of a microarray align to the genome (Figure 1) as some are non-specific with mismatches greater than one base pair whilst others have significant mapping to multiple locations (Figure S3).

While some probesets potentially target multiple genes (Figure 6), some probesets have been designed to specifically target a single gene. Sometimes these target a neighbouring gene as well and lead to ambiguous annotations. Stalteri et al examined in detail the problem of multiple probesets mapping to the same gene [37]. They specifically studied a single case, the Affymetrix MOE430 chip and the genes *Surf4*/*Surf2*, which are located in a tail-to-tail conformation on mouse chromosome 2 (Figure 9). For this microarray, Affymetrix identified eight probesets targeting the *Surf4 *gene. However, after careful alignments Stalteri et al found that five probesets actually target the *Surf4 *gene, two probesets target the gene *Surf2 *on the opposite strand, and one probeset maps to a different chromosome. For these eight probesets, Ensembl essentially replicates the manual annotation of Stalteri et al. We correctly annotate the five probesets to *Surf4 *and the probeset found to be targeting chromosome 19. The remaining two probesets, which Stalteri et al annotate to *Surf2 *are mapped in the same genomic location by our procedure, but we annotate only one of the two probesets to *Surf2*. This difference is due to Stalteri et al's reliance of an alternative splice acceptor site on exon 6, where one of the probeset mapped. This alternative splice site is not included in the current Ensembl gene set because is not supported by the protein evidence we used.

Some studies [6,36] have brought probeset mapping into another level in redefining complete probesets based on where probes aligned on transcripts. New probeset definitions regroup probes that consistently map to a set of transcripts or a single transcript variant. In their example with the probeset "33631_at" from HG_U95Av2, Lu, et al demonstrates how a set of 16 probes could be redefined in two distinct probesets, a probeset of 9 probes and another of 7 [36]. Their probeset redefinition is then be used to measure specific transcript differential expression. The Ensembl probeset annotation pipeline does not produce probeset redefinitions, but use the manufacturer's original design. However, using the Ensembl API, researchers could use the genomic probe mapping information and the Ensembl transcript predictions to redefine probes into new custom probesets.

While this paper focused on the Affymetrix GeneChip probesets to describe our procedure to map and annotate gene expression arrays, our pipeline is also implemented to annotate arrays made by other manufacturers, including both Illumina and CodeLink expression arrays.

## Conclusions

Our method to map microarray probes and annotate probesets to the latest Ensembl gene sets provides a consistent annotation of gene expression arrays available to researchers. For some arrays a substantial number of probesets cause problems but these issues have been identified and are taken into account by our annotation methods. However, with the recent advances in functional elements identification and the massive parallel sequencing technologies our understanding of the genome and transcriptome is evolving. Consequently, regularly updating our probe set annotation based on the latest genome assembly and gene predictions will continue to improve the analysis and understanding of gene expression arrays.

## Methods

### Probe mapping procedures

The Affymetrix GeneChip probe sequences are freely available on the Affymetrix website, and were downloaded for all the species included in Ensembl. Probes, targets, annotation, technical documentation files and others documents are available from the Affymetrix microarray product webpage http://www.affymetrix.com. For each species the probes supplied by Affymetrix are "collapsed" into a non-redundant set of probes because a single probe sequence can be a part of several array designs. The input to this step is the complete set of PM probes for all arrays of a given species. The output is a set of probes written into the oligo_array and oligo_probe tables in the appropriate Ensembl databases and a FASTA file of non-redundant probe sequences. In the alignment step the FASTA file of non-redundant PM probes generated in the last step is aligned against the corresponding genomic sequence files in softmasked-dusted fasta format. We use Exonerate [17] to align the probes against the genome, alignment must be exact or contain 1 mismatch at most, and probes should not align to the genome more than 100 times. Exonerate 1.4 is the current version used in our pipeline with the following options: --*bestn 100 --dnahspthreshold 116 --fsmmemory 256 --dnawordlen 14 --dnawordlimit 11*, and is available at http://www.ebi.ac.uk/~guy/exonerate/.

### Annotation to transcript

The annotation method associates microarray probesets with Ensembl transcripts. At least 50% of the PM probes in a probeset need to match the underlying transcript cDNA sequence for successful transcript annotation. In cases where a transcript has a 3' UTR predicted based on experimental evidence we use the transcript and twice the length of the 3' UTR sequence for the probeset annotation, otherwise we extend the 3' most exon. The length of the extension is precomputed as the longer of the median or the mean of all 3'UTR of the corresponding species. In the future, this extension will be adjusted to avoid any overlap with a gene in the proximity of the extended transcript.

### Mapping probes to cDNA

GeneChips probe sequences were aligned against cDNA sequences with Exonerate and using the same parameters as our current pipeline. For each transcript cDNA, sequences were downloaded from the corresponding Ensembl databases (Ensembl release 51) using the Perl API. For each alignment, coordinates were projected to genomic coordinates and probes mapping to exon-exon boundaries where kept for the analysis.

### Unannotated probesets genomic locations

For each unannotated probeset the genomic location of probes were obtained using the Ensembl API. The probe locations are computed at the transcript level, so for example a single probe hit could map to an exon on one transcript, and an intron on another transcript. For each unannotated probeset, the number of probes assigned to a category (exon, intron, 5'UTR, 3'UTR, and borders) is divided by the number of associated transcripts the given probeset. Finally, for each category, each ratio is centred by the sum of all ratios. For the ternary diagrams, locations were classified into three groups, the "non coding" group including intergenic region and intron categories, the "coding" group including the exon and 5'/3' UTR categories and finally the "border" group which includes probes located on border of one of these regions. Border locations are associated with the most 5' region, for example a probe spanning an exon-intron boundaries will be defined as being in the exon border, while a probe spanning an intron-exon will be defined as intron border.

### Comparison with Affymetrix annotation

All Affymetrix annotation files corresponding to the GeneChips included in our pipeline are publicly available and were downloaded from the Affymetrix website. All Human, Mouse, Rat but also C. elegans, Drosophila, Zebrafish, Rhesus, Chicken, Canine, Cow, Yeast and Anopheles Plasmodium GeneChips were annotated on 08/07/2008, except for MG_U74A/B/C annotated on 31/05/2007. The Affymetrix annotations for the RGD database are missing from the 19/03/2008 and 08/07/2008 annotation files, thus Affymetrix RGD annotation for the rat GeneChip have been replaced by the previous annotations files (06/11/2007). External accession numbers given by the Affymetrix annotations files were used to compare with our annotations. For each GeneChip, external identifiers associated with transcript-level annotated probesets were extracted using the Ensembl Perl API and compared with Affymetrix external annotations. When Affymetrix or Ensembl provided more than one external identifier for the same probeset and the same database, one accession identifier in common was enough to connect the two annotations.

### Probesets annotated to multiple genes

Genes linked to same probeset where extracted and analysed for homologous or overlapping evidence. The "within_species_paralogues" inferred from gene trees were extracted from the Ensembl Compara 51 database using the Ensembl Compara API [38]. A within-species paralog corresponds to a relation between two genes of the same species where the ancestor node has been labelled as a duplication node. The duplication event could have occurred in this species only, or at a different duplication time depending on the ancestor node taxonomy level. Overlapping genes are defined as two tandem genes having their start/end overlapping after UTR extension.

### Allele-specific probes and mismatches

For each probe mapping we looked for the presence of one or more SNPs within that region regardless of the presence of a mismatch in the alignment. Species-specific Ensembl Variation release 51 databases and the variation Perl API were used to obtain the SNPs available for each probe location. Probes with SNPs found in their genomic location where categorized in four groups: the mismatch with or without SNPs groups, and the perfect match with or without SNPs groups. The mismatch groups correspond to probes mapping the genome with one mismatch as defined in the mapping procedures.

### Public access

The Ensembl API allows programmatic access to all of the arrays reannotated by our pipeline. The probe mapping and probeset annotation data can be directly accessed using our public MySQL servers (host: ensembldb.ensembl.org, user: anonymous, port: 5306 for Ensembl releases above 48). We also provide a Perl API to programmatically access the mapping and annotation in our database as well as all other data available in Ensembl. Documentation schema descriptions, tutorials and also instructions on how to download and install the Ensembl APIs (Core, Compara, Variation, Functional Genomics) can found at http://www.ensembl.org/info/data/api.html. An example script to extract probes mapping and probesets annotation is available as an additional file (Additional file 5). BioMart can be accessed directly from the BioMart central server http://www.biomart.org or from http://www.ensembl.org/biomart/martview/ for the Ensembl databases. Biomart provides an intuitive web interface to access our data. Finally, the R package biomaRt is available through BioConductor, some external packages (XML, RCurl) are required for installation.

## Authors' contributions

BB was responsible for designing and implementing the analyses, interpreting the data. NJ and GP contribute to the Ensembl software developments. NJ develops and maintains the probeset annotation pipeline and produces the data. The manuscript was written by BB, NJ, GP and PF. PF conceived the study, coordinated the study and the writing. All authors read and approved the final manuscript.

## Supplementary Material

Additional file 1 **Distribution of probes mapped to genomes**. Diamonds represents distribution of the numbers probes per number of hits. These are the numbers of all mapped probes per number of mappings once the mapping rules are applied for all human (red), mouse (blue), and rat (black) arrays. The values on the y-axis are the log scaled counts of probes for a number of hits on the genome. The crosses represent the percentage of probes targeting repeat regions, y-axis on the left. Click here for file

Additional file 2 **Probes mapping to multiple exons boundaries**. Example of a probe being aligned on three exons, where the sequence in red corresponds to the probe and blue/black sequences correspond to exons. Click here for file

Additional file 3 **Genomic distribution of unannotated probesets in EST genes**. In contrast to our protein based gene prediction methods, Ensembl predicts EST genes and transcripts by using evidence from ESTs only [27]. As represented by the figure below some of these unannotated probesets could also be linked to EST predicted genes. However, EST gene predictions often overlap with protein coding predictions thus probesets being linked to an EST gene do not necessarily mean a probeset annotation is missing. For example, the un-annotated probeset 1439918_at for the Mouse430_2 array has probes mapping to 6 EST transcripts from the EST gene ENSMUSESTG00000015935, but this probeset has also probes mapping to almost an identical protein-coding gene ENSMUSG00000026790. On the ternary graph, the mappings of un-annotated probesets are almost identical between the protein coding genes and the EST genes with clear concentration of probesets being mapped in non-coding regions. On the other hand, probesets with probes located in the coding regions have less than half of their probes matching the underlying transcript (blue dots) and thus do not satisfy our annotation rules. Click here for file

Additional file 4 **Comparison of Ensembl and Affymetrix annotations**. Affymetrix annotations are compared with Ensembl annotations using external database identifiers cross referenced to the Ensembl gene predictions. For a given external database the percentage of common annotation between Ensembl and Affymetrix are shown. Click here for file

Additional file 5 **Example Perl script using the Ensembl API**. This Perl script extracts probes mapping and probeset annotations for the Mouse Affymetrix array MOE430A, on the chromosome 2 between coordinates 26771920 and 26789448. Click here for file
