# Supplementary material for: Consistent annotation of gene expression arrays
Source: BMC Genomics. 2010 May 11;11:294. doi: 10.1186/1471-2164-11-294 (PMC2894801; doi:10.1186/1471-2164-11-294)
Supplement: Additional file 1 — Distribution of probes mapped to genomes. Diamonds represents distribution of the numbers probes per number of hits. These are the numbers of all mapped probes per number of mappings once the mapping rules are applied for all human (red), mouse (blue), and rat (black) arrays. The values on the y-axis are the log scaled counts of probes for a number of hits on the genome. The crosses represent the percentage of probes targeting repeat regions, y-axis on the left. [file 1471-2164-11-294-S1.PDF]

Supplement 1

Distribution of probes mapped to genomes.

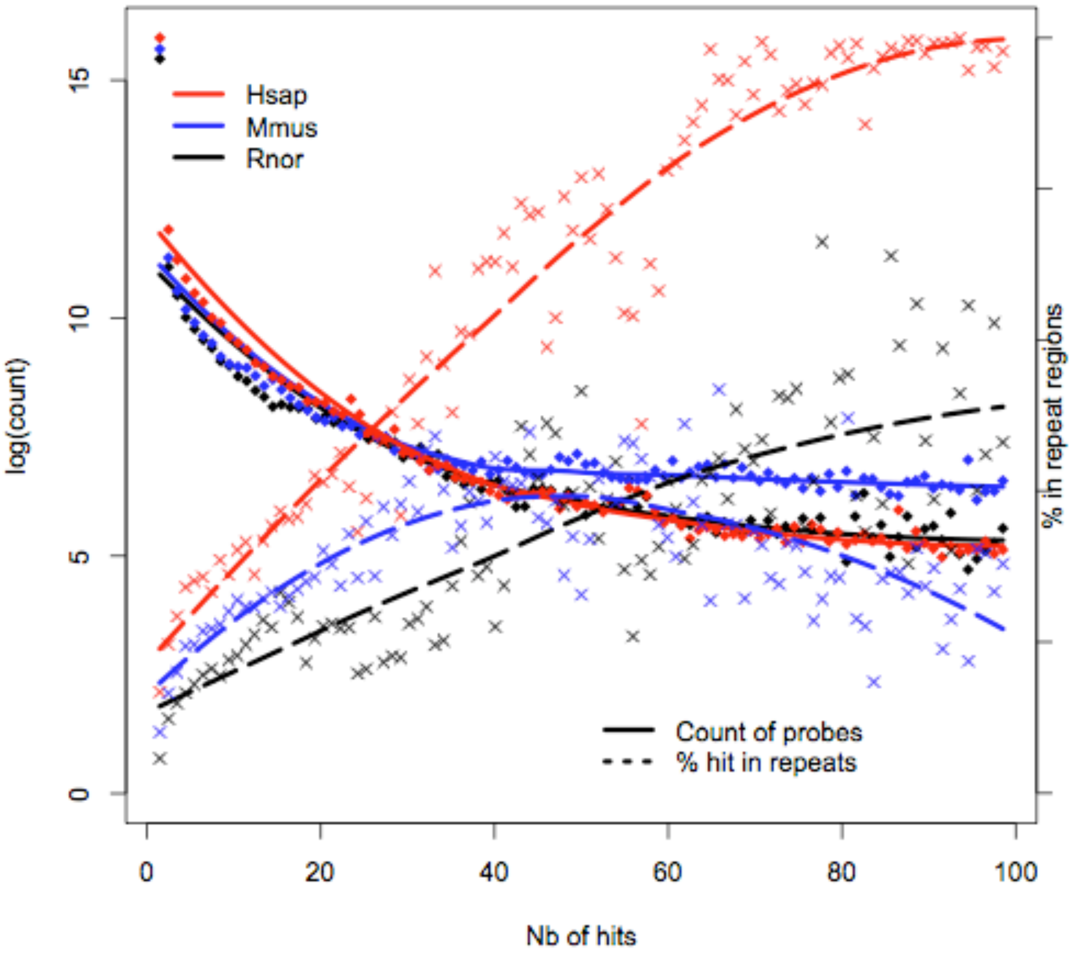

Diamonds represents distribution of the numbers probes per number of hits. These are the numbers of all mapped probes per number of mappings once the mapping rules are applied for all human (red), mouse (blue), and rat (black) arrays. The values on the y axis are the log scaled counts of probes for a number of hits on the genome. The crosses represent the percentage of probes targeting repeat regions, y axis on the left.
