# Supplementary material for: Consistent annotation of gene expression arrays
Source: BMC Genomics. 2010 May 11;11:294. doi: 10.1186/1471-2164-11-294 (PMC2894801; doi:10.1186/1471-2164-11-294)
Supplement: Additional file 2 — Probes mapping to multiple exons boundaries. Example of a probe being aligned on three exons, where the sequence in red corresponds to the probe and blue/black sequences correspond to exons. [file 1471-2164-11-294-S2.PDF]

```
>ENST00000307959__probe:HG-U133_Plus_2:207136_at:1089:1005;
ENST GAACTTCATCTCTCTCCCCAGAAAAGGCTCAACATCAACTATATAGCCAACATGTCCAAG
Prob ............................................................

ENST GTGTTTAAGAAGACCAGCTCCAATGGGAAGCTCTCCATCTACCTGGGGAAACGGGACTTC
Prob ............................................................

ENST GTGGACCATGTGGACACGGTGGAACCCATTGACGGTGTTGTCCTGGTTGATCCTGAGTAC
Prob ............................................................

ENST TTAAAATGTCGAAAGTTGTTTGTCATGTTGACATGTGCCTTTCGCTATGGCCGTGATGAC
Prob ............................................................

ENST TTGGAAGTGATTGGTCTGACGTTCCGAAAAGATCTGTATGTGCAGACCCTGCAAGTGGTC
Prob ............................................................

ENST CCAGCTGAATCCAGCAGCCCTCAGGGGCCCCTCACAGTCCTACAGGAGCGACTACTGCAC
Prob ............................................................

ENST AAGCTAGGGGACAATGCCTACCCCTTTACCCTGCAGATGGTGACCAACCTGCCCTGTTCT
Prob ............................................................

ENST GTGACACTGCAGCCAGGTCCTGAAGATGCAGGAAAGCCCTGTGGGATTGACTTTGAAGTG
Prob ............................................................

ENST AAGAGTTTCTGTGCTGAAAACCCAGAGGAGACAGTCTCCAAGAGAGACTATGTGCGGCTG
Prob ............................................................

ENST GTTGTCCGGAAAGTACAATTTGCACCACCGGAGGCAGGCCCTGGCCCCTCAGCCCAGACC
Prob ............................................................

ENST ATCCGCCGCTTCCTTCTGTCAGCTCAGCCCCTACAACTCCAGGCCTGGATGGACAGGGAG
Prob ............................................................

ENST GTTCACTACCACGGAGAACCCATCTCTGTCAATGTTTCTATCAACAACTGCACCAACAAG
Prob ............................................................

ENST GTCATCAAAAAAATCAAGATTTCAGTTGACCAGATCACAGATGTTGTCCTGTATTCACTA
Prob ............................................................

ENST GACAAGTACACCAAGACTGTGTTCATTCAGGAATTCACGGAGACTGTAGCTGCTAATTCC
Prob ............................................................

ENST AGCTTCTCCCAGAGCTTTGCAGTAACCCCAATCCTGGCTGCCAGCTGCCAGAAACGGGGC
Prob ............................................................

ENST CTGGCACTGGATGGCAAACTTAAGCATGAAGATACCAACCTGGCCTCTAGCACAATTATT
Prob ............................................................

ENST AGACCGGGAATGGACAAAGAGCTGCTGGGGATCCTGGTGTCCTACAAAGTCAGAGTCAAC
Prob ............................................................

ENST CTGATGGTGTCCTGTGGTGGCATCCTAGGAGACCTGACAGCCAGCGATGTTGGTGTGGAG
Prob ............................................................

ENST CTACCCTTGGTCCTGATCCATCCGAAGCCATCTCATGAGGCCGCTAGCTCTGAGGACATA
Prob ..............................TCTCATGAGGCCGCTAGCTCTGAGG.....

ENST GTCATCGAGGAGTTTACGCGGAAAGGCGAGGAGGAGAGCCAGAAGGCTGTGGAGGCTGAG
Prob ............................................................

ENST GGAGATGAGGGGAGCTGA
Prob ..................
```
