# Supplementary material for: Consistent annotation of gene expression arrays
Source: BMC Genomics. 2010 May 11;11:294. doi: 10.1186/1471-2164-11-294 (PMC2894801; doi:10.1186/1471-2164-11-294)
Supplement: Additional file 3 — Genomic distribution of unannotated probesets in EST genes. In contrast to our protein based gene prediction methods, Ensembl predicts EST genes and transcripts by using evidence from ESTs only [27]. As represented by the figure below some of these unannotated probesets could also be linked to EST predicted genes. However, EST gene predictions often overlap with protein coding predictions thus probesets being linked to an EST gene do not necessarily mean a probeset annotation is missing. For example, the un-annotated probeset 1439918_at for the Mouse430_2 array has probes mapping to 6 EST transcripts from the EST gene ENSMUSESTG00000015935, but this probeset has also probes mapping to almost an identical protein-coding gene ENSMUSG00000026790. On the ternary graph, the mappings of un-annotated probesets are almost identical between the protein coding genes and the EST genes with clear concentration of probesets being mapped in non-coding regions. On the other hand, probesets with probes located in the coding regions have less than half of their probes matching the underlying transcript (blue dots) and thus do not satisfy our annotation rules. [file 1471-2164-11-294-S3.PDF]

Supplement 3

Genomic distribution of unannotated probesets in EST genes.

In contrast to our protein based gene prediction methods, Ensembl predicts EST genes and transcripts by using evidence from ESTs only [22]. As represented by the figure below some of these unannotated probesets could also be linked to EST predicted genes. However, EST gene predictions often overlap with protein coding predictions thus probesets being linked to an EST gene do not necessarily mean a probeset annotation is missing. For example, the un-annotated probeset 1439918_at for the Mouse430_2 array has probes mapping to 6 EST transcripts from the EST gene ENSMUSESTG00000015935, but this probeset has also probes mapping to almost an identical protein-coding gene ENSMUSG00000026790. On the ternary graph, the mappings of un-annotated probesets are almost identical between the protein coding genes and the EST genes with clear concentration of probesets being mapped in non-coding regions. On the other hand, probesets with probes located in the coding regions have less than half of their probes matching the underlying transcript (blue dots) and thus do not satisfy our annotation rules.

EST genes

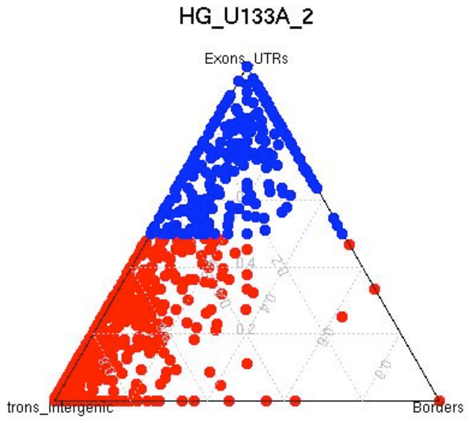

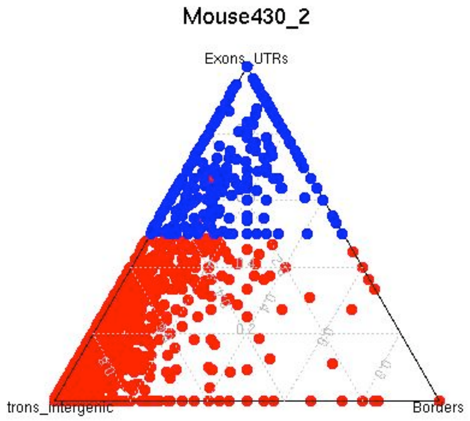

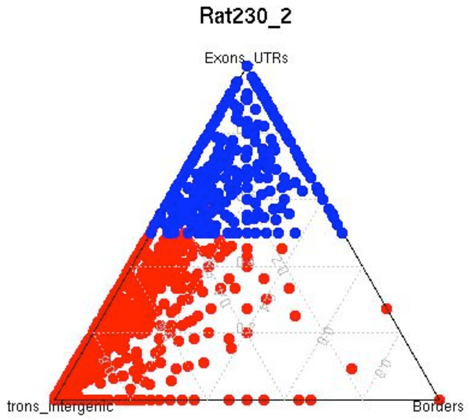

Genomic distribution of unannotated probesets in EST genes.
